# Prediction and Activity of a Cationic α-Helix Antimicrobial Peptide ZM-804 from Maize

**DOI:** 10.3390/ijms22052643

**Published:** 2021-03-05

**Authors:** Mohamed F. Hassan, Abdelrahman M. Qutb, Wubei Dong

**Affiliations:** 1 The Key Lab of Crop Disease Monitoring and Safety Control in Hubei Province, Department of Plant Pathology, College of Plant Science and Technology, Huazhong Agricultural University, Wuhan 430070, China; Mohamedzaki@webmail.hzau.edu.cn (M.F.H.); a.m.qutb@azhar.edu.eg (A.M.Q.); 2 Department of Agriculture Botany, Faculty of Agriculture, Al-Azhar University, Cairo 11651, Egypt

**Keywords:** antimicrobial peptide, maize, AMP prediction, CAMP_R3_ database, bacteria pathogens, plant protection

## Abstract

Antimicrobial peptides (AMPs) are small molecules consisting of less than fifty residues of amino acids. Plant AMPs establish the first barrier of defense in the innate immune system in response to invading pathogens. The purpose of this study was to isolate new AMPs from the *Zea mays* L. inbred line B73 and investigate their antimicrobial activities and mechanisms against certain essential plant pathogenic bacteria. In silico, the Collection of Anti-Microbial Peptides (CAMP_R3_), a computational AMP prediction server, was used to screen a cDNA library for AMPs. A ZM-804 peptide, isolated from the *Z. mays* L. inbred line B73 cDNA library, was predicted as a new cationic AMP with high prediction values. ZM-804 was tested against eleven pathogens of Gram-negative and Gram-positive bacteria and exhibited high antimicrobial activities as determined by the minimal inhibitory concentrations (MICs) and the minimum bactericidal concentrations (MBCs). A confocal laser scanning microscope observation showed that the ZM-804 AMP targets bacterial cell membranes. SEM and TEM images revealed the disruption and damage of the cell membrane morphology of *Clavibacter michiganensis* subsp. michiganensis and *Pseudomonas syringae* pv. tomato (Pst) DC3000 caused by ZM-804. In planta, ZM-804 demonstrated antimicrobial activity and prevented the infection of tomato plants by Pst DC3000. Moreover, four virulent phytopathogenic bacteria were prevented from inducing hypersensitive response (HR) in tobacco leaves in response to low ZM-804 concentrations. ZM-804 exhibits low hemolytic activity against mouse red blood cells (RBCs) and is relatively safe for mammalian cells. In conclusion, the ZM-804 peptide has a strong antibacterial activity and provides an alternative tool for plant disease control. Additionally, the ZM-804 peptide is considered a promising candidate for human and animal drug development.

## 1. Introduction

Antimicrobial peptides (AMPs) are short peptides, usually among 12 and 50 amino acids (aa) in length, with broad-spectrum activities against microorganisms. Historically, AMPs are known as host-defense peptides, cationic amphipathic peptides, cationic-host defense peptides [1], and alpha-helical antimicrobial peptides [2]. Host defense peptides (HDPs) indicate the vital role of AMPs in preventing most disease infections before any symptoms are induced [3]. Plants’ AMPs are the first barrier defense component in the innate immune system; they are usually generated upon attack by microorganisms and play a vital role in the innate immune system [4,5]. AMPs are small peptides, mostly forming an alpha-helix or β sheet structure, and they confer protection against fungi and bacteria infections as well as certain viruses [6]. Plant genomes hold a wide range of genes for detecting pathogen attack and inducing adequate defense responses [7]. Several small basic peptides were identified from kernels of maize inbred line B73 and with antimicrobial properties [8]. Plant AMPs prevent and eliminate pathogens via penetration and disruption of cell membranes’ structural components [9]. Some AMPs from plants can be incorporated within the microbial cell membrane and create pore-like gaps that cause essential nutrient and ion leakage, eventually leading to microbial cell death [10]. The sequences of AMPs identified to date are available from several web databases such as PhytAMP and UniProt which provide details about AMP biological data and sequences. Additionally, bioinformatics analysis provides a tool to increase our understanding of AMPs [11]. In recent years, emerging AMPs from plants have become a huge challenge to predict their antimicrobial activities against pathogens using computational methods that utilize several algorithms based on strictly defined parameters. In silico discovery and prediction of AMPs relies on the well-developed AMP databases, which has resulted in discovering new AMPs such as PhytAMP in plants [12]. Several in silico resources have integrated a wide range of AMPs for many species, such as the Collection of Anti-Microbial Peptides (CAMP_R3_) which presently holds data of 10,247 AMP sequences, 757 structures, and 114 AMPs family-specific signatures related to AMP data analysis [13]. Another reliable resource is APD3: a thorough peptide detection database with mostly documents natural peptides that established antimicrobial activity. The APD3 also offers additional information that includes physicochemical properties, composition, characteristics structure of amino acids, classification, nomenclature, calculations, design, and AMP prediction [14]. The dbAMP, ClassAMP, iAMPpred, and AntiBp are prediction and classification tools based on peptide sequences for predicting AMP function and the type of the peptide as antifungal, antibacterial, or antiviral agents [15]. Alpha-helical peptides are responsible for about 50% of the known AMPs, and amphipathicity is regarded as a requirement for α-helical AMPs activity [16]. Several studies have shown that peptide helicity can be more critical than the antimicrobial activity of AMPs for toxicity [17]. In nature, AMPs interact with the membrane, assuming that an amphipathic α-helical configuration of the peptide is obtained and a well-defined hydrophobic region is inserted into the lipid bilayer. The activity of AMPs depends on several essential parameters, including the α-helical structure, secondary structure, amphipathicity, cationic at least +2, and hydrophobicity, as well as sequence and molecular weight [18,19]. The determination of minimal inhibitory concentration (MIC) and minimum bactericidal concentration (MBC) is recommended by the Clinical and Laboratory Standards Institute (CLSI) to study the activity of the peptides against microbes [20]. The cationic AMPs (positive charge) interact with the bacterial cell’s anionic membrane (negative charge), which has a disruptive effect on the pathogenic bacteria (Gram-positive and Gram-negative) [21]. Cationic AMPs target the cell wall peptidoglycan in Gram-positive bacteria or the outer membrane of lipopolysaccharide (LPS) in Gram-negative bacteria as a process of a key step in agglutinations [22]. Phytopathogenic bacteria (i.e., *Pseudomonas*, *Xanthomonas*, *Corynebacterium*, *Erwinia*, and *Agrobacterium*) cause many plant bacterial diseases and their symptoms such as leaf spot, streak, wilt, blight, rot, canker, and galls [23,24]. They impose great economic losses of crops and represent a huge challenge to control and prevent. The major control of plant diseases is dependent on chemical pesticides; however, the impact of long-term ecological pollution and carcinogenic effects on humans and other animals signify the need to find safer alternatives [25].

In this study, we aimed to (a) isolate and characterize new AMPs from *Z. mays* L. inbred line B73, (b) investigate their antimicrobial activities against some critical plant pathogenic bacteria to manage and improve the prevention of plant diseases, and (c) to explore the mode of action of peptides that underlies this antimicrobial activity.

## 2. Results

### 2.1. cDNA Library of Maize B73

cDNA inserts from *Z. mays* B73 were cloned into *Bacillus subtilis* SCK_6_ in order to construct the cDNA library. During this process, around 2000 single colonies were individually collected. Colony PCR results revealed that most of the bands of inserts consisted of at least 500 bp, which reflects the high quality of the cDNA library (Figures S1 and S2). cDNA insert sequences were investigated for both identification and similarity with other published sequences using the Basic Local Alignment Tool (BLAST) in the National Center for Biotechnology Information (NCBI) and the Maize Genetics and Genomics Database (MaizeGDB).

### 2.2. Bioinformatics Analysis

#### 2.2.1. In Silico Prediction for AMPs

The cDNA inserts were translated into amino acid sequences using a server translation. Upon completion, sequences were screened for AMPs using the CAMP**_R3_** prediction server (SVM, RF, DA, and ANN algorithms). As a result of AMPs prediction, 14 amino acid sequences were predicted as AMPs with prediction scores higher than 0.5. The ZM-804 peptide showed the uppermost prediction score among the 14 sequences of interest, which is considered the most potential antimicrobial peptide candidate. Moreover, the ADAM server (utilizing the SVM algorithm) predicted the ZM-804 as AMP with a score of 1.34 (Table S4). Furthermore, all predictions of the ZM-804 peptide using dbAMP, ClassAMP, iAMPpred, and AntiBp servers were positive and showed potential antibacterial activity (Table S5).

#### 2.2.2. Physiochemical Characterization of the ZM-804 Peptide

**Figure 1:**
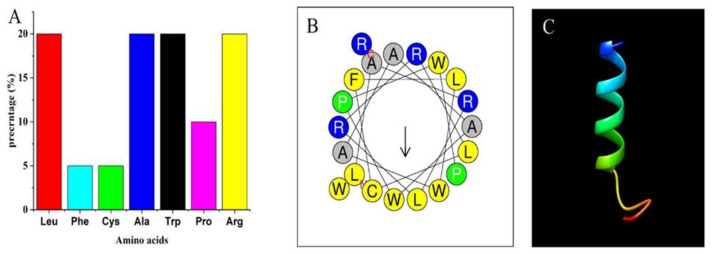
Bioinformatics analysis of the ZM-804 peptide. (**A**) Amino acids composition of the ZM-804 showing the highest percentage in hydrophobicity (diagram created by Origin^®^). (**B**) In the helical wheel diagram of the ZM-804 peptide, the positively charged residues are defined by blue color while the hydrophobic residues are defined by yellow and grey. The ZM-804 peptide showed confirmations of an amphipathic alpha-helix. (**C**) 3D structure of the ZM-804 predicted by the I-TASSER and visualized by the Chimera 1.14rc software.

Physicochemical properties of the ZM-804 peptide were determined based on deep sequence analysis of the peptide. The APD3-based prediction indicated that the ZM-804 consists of a short peptide sequence containing 20 amino acids, which originated from seven different groups of amino acids (leucine 4 (20%), phenylalanine 1 (5%), cysteine 1 (5%) alanine 4 (20%), tryptophan 4 (20%), proline 2 (10%), and arginine 4 (20%)) (Figure 1A). According to the APD3, a physicochemical property of the ZM-804 was predicted as an alpha-helix peptide based on the peptide sequence. Additionally, the APD3 revealed some key AMP properties: (a) the high hydrophobic ratio of 70%, positive net charge +4, and (b) at least five residues on the same hydrophobic surface. Previous findings are considered strong indicators that the ZM-804 may interact with the cell membranes. Therefore, the ZM-804 is a candidate of AMP. The Wimley–White whole residue hydrophobicity parameter was predicted to be -6.19 kcal/mol (a more hydrophobic peptide tends to have a more negative value, while a more hydrophilic peptide tends to have a more positive value), and the theoretical isoelectric point (pI) was 12.288. The helical wheel illustration of the ZM-804 peptide was predicted as an amphipathic alpha-helix (Figure 1C), and most of the hydrophobic residues are in the opposite position of the alpha-helix. However, some of the hydrophobic residues F, W, L, and two Ala, are placed along with the cationic residues. The hydrophobic helical moments mean (<μH>) is 0.403, the hydrophobicity (<H>) is 0.888, and net charge (z) 4 were determined using the Heliquest analysis server (Figure 1B). The discrimination factor (D) of the ZM-804 peptide was 1.7, which predicted it as a potential lipid-binding peptide (Table 1). The secondary structure of the ZM-804 peptide was predicted by the I-TASSER server and showed that the ZM-804 peptide has an alpha-helix structure. The secondary structure and helical wheel modeling prediction proved that the hydrophilic and the hydrophobic residues are sited on opposite sides of the alpha-helix structures of the ZM-804 peptide, and an amphipathic alpha-helix was predicted. The ZM-804 peptide sequence showed no significant similarities to any AMPs in the CAMP_R3_, MLAMP, DRAMP, dbAMP, APD3, and ADAM databases. Consequently, as a result of BLAST, there were no similarities between the ZM-804 peptide and other sequences of AMPs database tested, which confirms the novelty of the ZM-804 peptide as an AMP.

#### 2.2.3. The 3D Structure of ZM-804

The I-TASSER and the PEP-FOLD3 servers were used to predict the model of the alpha-helical 3D structure of ZM-804 (Figure 1C). The best structural model of the ZM-804 peptide was chosen by the PROSAII and the MolProbity tools to avoid any mistakes. The ZM-804 peptide was revealed to be a helical structure within the practical quality ranges. Z-score values obtained by the PROSAII software, which suppose that most points of the data in the multidimensional NMR spectrum are at the position not occupied via signals [26], showed the ZM-804 peptide was within the appropriate region of structures, propped that it has characteristics of the native structure [27]. The MolProbity reported that the percentage of ZM-804 peptide residues had all the residues within the favorable region by Ramachandran favored 94.44%, Ramachandran outliers 5.56%, and poor rotamers 0%, suggesting that the parameters of the ZM-804 structure remain within the ranges of acceptable consistency and stability [28].

### 2.3. Antimicrobial Assay of the ZM-804 Peptide

#### 2.3.1. The MIC and MBC of the ZM-804 Peptide

The antimicrobial activity of the ZM-804 against 11 indicators of Gram-positive and Gram-negative phytopathogenic bacteria along with one model strain of *B. subtilis* and two strains of *E. coli* has been investigated. The MICs and MBCs assays were evaluated for all of the bacterial indicators tested. The results showed that the lowest concentration of the ZM-804 peptide inhibited 80% growth of bacteria, compared to the control. The lowest MIC values determined were 8 and 4 µM for Gram-positive and Gram-negative bacteria, respectively, while the highest value was 16 µM for Gram-positive and Gram-negative bacteria. The MBC value in response to ZM-804 peptide concentrations was determined; the lowest concentration, which led to no visible growth of bacteria (MBC) was 16 and 8 µM for Gram-positive and Gram-negative bacteria, respectively. In comparison, the highest values were 32 and 16 µM for Gram-positive and Gram-negative bacteria, respectively (Table 2).

#### 2.3.2. The MLC of the ZM-804 Peptide

**Figure 2:**
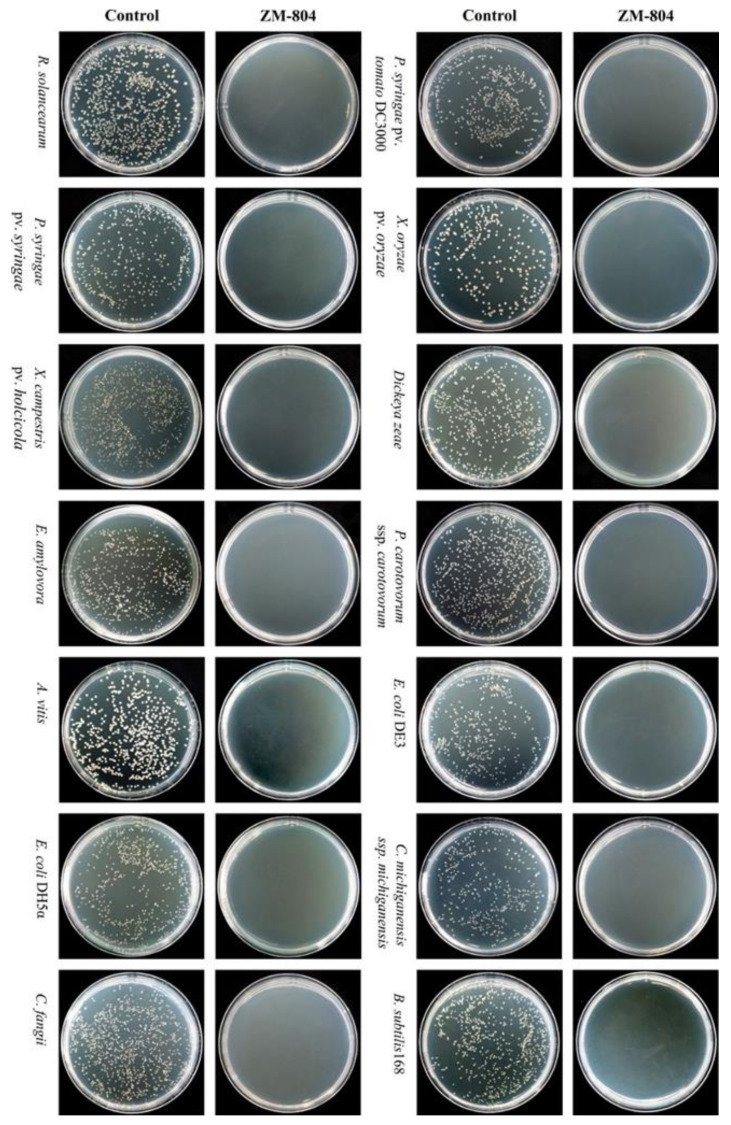
The MLC of the ZM-804 peptide. No bacterial growth was observed after treatment with 4 µM ZM-804 peptide for all bacterial indicators (Gram-positive and Gram-negative). In contrast, bacterial indicators have grown normally on the control plates (treated with dd water).

The minimal lethal concentration (MLC) of the ZM-804 peptide which caused the complete death of bacteria was determined against all bacterial indicators (Gram-positive and Gram-negative bacteria). The concentration value of MLC for all bacterial indicators was observed after treatment by serial dilutions (128, 64, 32, 16, 8, 4, 2, and 1 µM) of the ZM-804 peptide in the plates that did not show any growth of the colony (complete death). In contrast, bacterial growth within control plates for all bacterial indicators was observed. Thus, ≤4 was considered the lowest ZM- 804 concentration, which caused complete death of all bacterial indicators (Figure 2).

### 2.4. Localization of FITC-Labeled ZM-804 Peptide 

**Figure 3:**
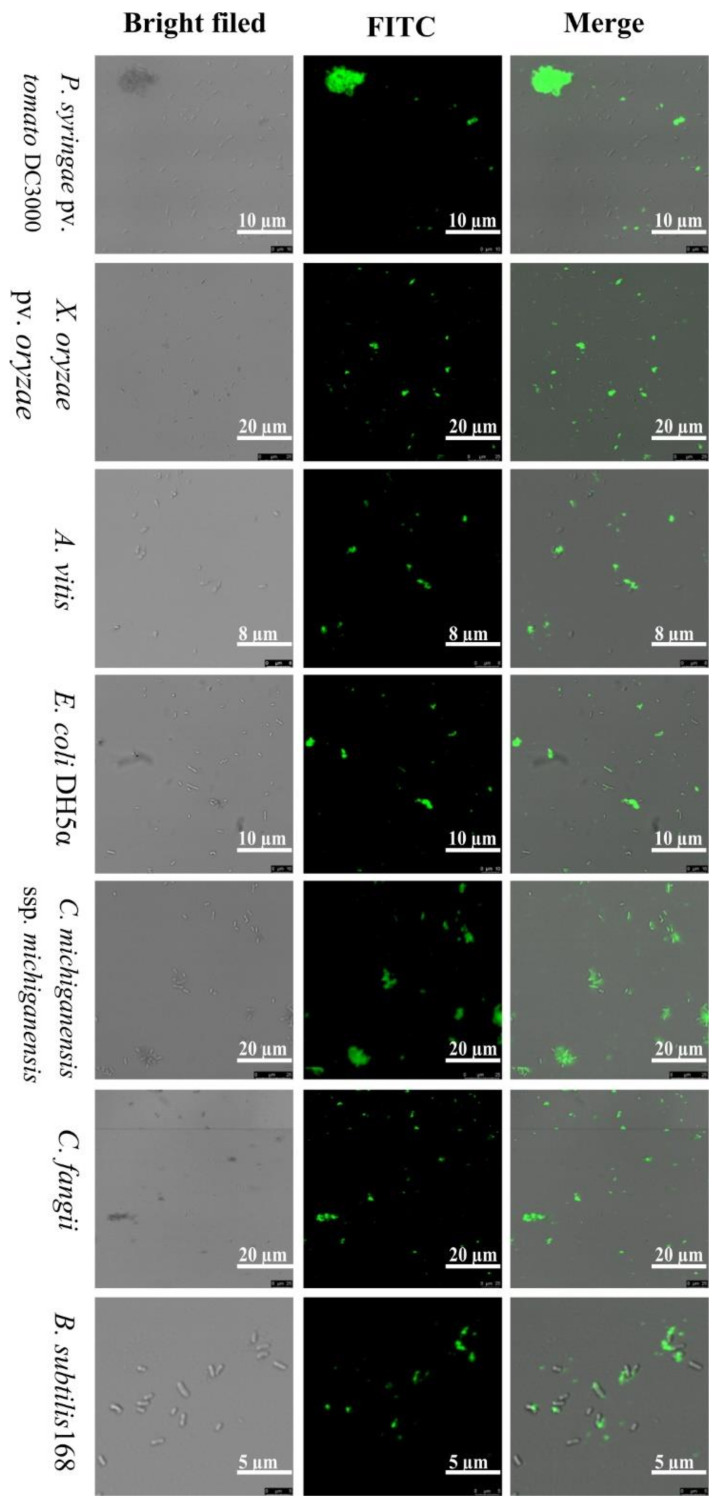
Localization of the FITC-ZM-804 peptide. Bacterial cells were treated with 10 µM of FITC-labeled ZM-804 peptide for 4 h and visualized under an Olympus BX61 confocal laser scanning microscopy. The localization of the ZM-804 peptide was indicated by the green fluorescence due to the interaction between ZM-804 and the cell membrane of bacterial indicators (Gram-positive and Gram-negative bacteria).

To evaluate the mechanism of the ZM-804 as an AMP and the interaction with the bacterial cell plasma membrane, 10 µM of the Fluorescein isothiocyanate (FITC) labeled-ZM-804 peptide was incubated for 4 h with the bacterial indicator and their localization visualized via an Olympus BX61 confocal laser-scanning microscope. All bacterial indicators, Gram-positive bacteria (*C. michiganesis* subsp. michiganesis, *C. fangii*, and *B*. *subtilis* 168), and Gram-negative bacteria (*Ralstonia solanacearum, P. syringae* pv. tomato DC3000, *P*. *syringae pv.* syringae, *Xanthomonas oryzae* pv. oryzae, *X. campestris* pv. holcicola, *Dickeya zeae*, *Erwinia amylovora*, *Pectobacterium carotovorum* subsp. carotovorum, *Agrobacterium vitis,* and *E. coli* DE_3_ and DH_5_α) have shown green fluorescence under the confocal microscope (Figure 3). As expected, the FITC-labeled ZM-804 peptide was attached and associated through the surface of bacterial cells. Previous results indicated that the major location of action is the bacterial cell membrane, which may confer ZM-804 the ability to penetrate the cell membrane and be distributed in the cytoplasm. To investigate the effect of the FITC tag on the ZM-804 peptide, the antimicrobial activity test was performed for four bacterial indicators (*X. campestris* pv. holcicola, *X. oryzae* pv. oryzae, *C*. *michiganesis* subsp. michiganesis, and *C. fangii*). All suspensions of bacterial indicators (~10^6^ CFU/mL) were treated with FITC-labeled-ZM-804 peptide and dd water control for 4 h. All of the control plates showed the colonies’ growth, whereas the plates of bacteria treated with FITC-ZM-804 did not detect any colony grown on plates of *C*. *michiganesis* subsp. michiganesis, *C. fangii*, *X. oryzae* pv. oryzae, and *X. campestris* pv. holcicola treated by 10 µM. As a result, the antimicrobial activity of the FITC-ZM-804 peptide decreased, which indicated that the FITC tag negatively affects ZM-804 peptide antimicrobial activity.

### 2.5. Effect of the ZM-804 Peptide on Bacterial Cell Membranes

**Figure 4:**
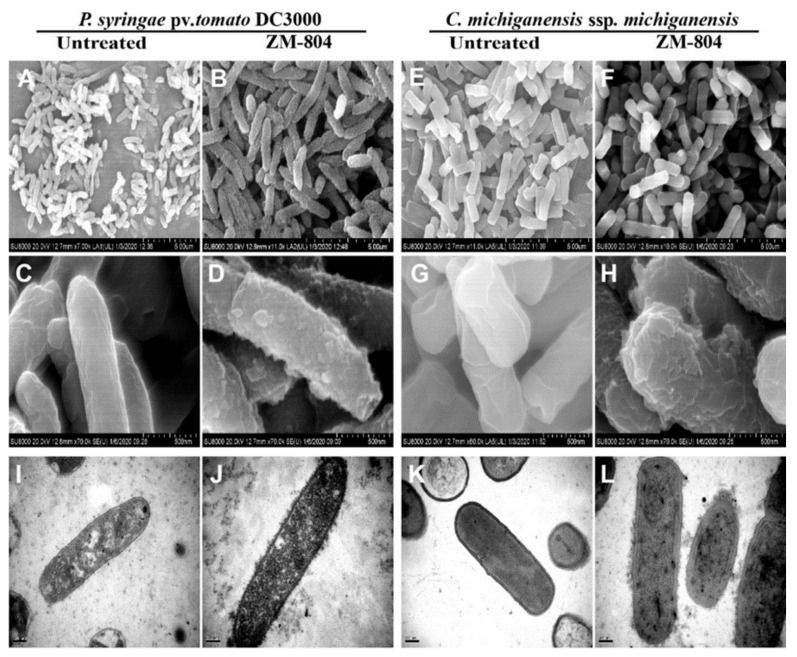
SEM and TEM investigations of *P*. *syringae* pv. tomato DC3000 and *C*. *michiganensis* subsp. michiganensis after treatment with ZM-804 peptide. Bacterial cells (∼10^7^ CFU/mL) were treated with 4 µM ZM-804 peptide for 4 h, while the control was treated with dd water. In control bacteria, SEM, and TEM images show that the cell membranes were intact without any cell damage (**A**,**C**,**E**,**G**,**I**,**K**). (**B**,**D**,**F**,**H**): The cell membrane of bacteria treated with ZM-804 was covered with bubbles and became rough. (**J**,**L**): This showed that the cell membrane was disrupted and substances remained present in the intercellular fluid. The results were obtained by the HITACHI SU810 for SEM and the HITACHI H-7650 for TEM.

SEM and TEM were used to observe the damage of the cell membrane (outer, inner) of *P. syringae* pv. tomato DC3000 and *C. michiganensis* subsp. michiganensis as representative Gram-positive and Gram-negative bacteria, respectively, upon treatment with the ZM-804 peptide. Both bacterial suspensions (~10^7^ CFU/mL) were treated by 4 µM of the ZM-804 peptide for 4 h, whereas dd water was added to untreated bacteria for the control treatment. SEM images for both bacteria treated with ZM-804 peptide showed several blisters and protruding bleb from the cell membrane surface of *P. syringae* pv. tomato DC3000 and irregular-wrinkled or corrugating with *C. michiganensis* subsp. michiganensis (Figure 4B,D,F,H). Generally, all the surfaces of bacterial cells were covered with bubbles after treatment with the ZM-804 peptide. In contrast, untreated bacterial cells (seen in rods) maintained intact, smooth, and bright surfaces without any damage to the cell membrane (Figure 4A,C,E,G).

TEM observations showed that untreated bacteria maintained intact well-defined membranes having cytoplasmic content distributed and filling the internal space well (Figure 4I,K). The cell wall-treated bacteria surface became wrinkled and rough, while the cytoplasmic content was not clear to distinguish (Figure 4J,L). The cell membranes (outer, inner) and cell walls of the bacteria were heavily affected, which was seen from the changes on the envelope of the bacterial cell and frequently covered all the bacteria cells, indicating that most of the deformation was caused by the ZM-804 peptide. Therefore, the ZM-804 peptide penetrates the cell membrane and increases the permeability of the cell membrane.

### 2.6. In Planta Antimicrobial Activity Assay of the ZM-804 Peptide

#### Hypersensitive Reaction (HR) on Nicotiana Benthamiana

**Figure 5:**
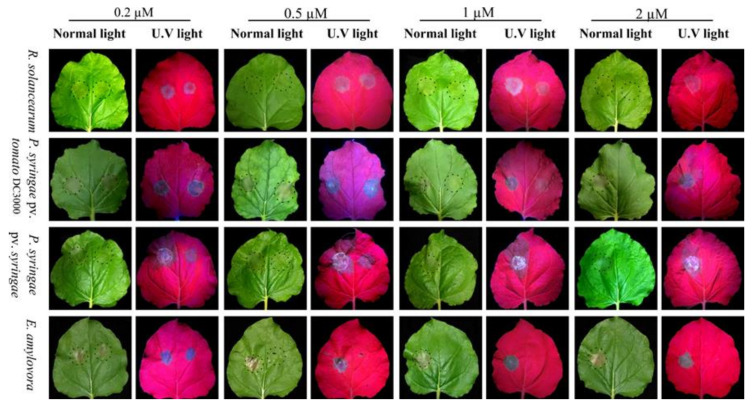
In planta antimicrobial activity assay on *N. benthamiana*. Bacterial cells (10^6^ CFU/mL) of *R. solanacearum*, *P. syringae* pv. tomato DC3000, *P*. *syringae* pv. syringae, and *E. amylovora* were treated with serial dilutions (0.2, 0.5, 1, and 2 µM) of ZM-804 peptide, while controls were treated with dd water for 4 h. In the infiltration method, the left side of the leaf blades was infiltrated with the control, while the right side was infiltrated with bacteria treated with ZM-804 peptide. The responses were photographed in normal and UV light 48 h after infiltration, respectively. The dotted circles represent infiltrated areas.

*R. solanacearum*, *P. syringae* pv. syringae, *P. syringae* pv. tomato DC3000, and *E. amylovora* virulence pathogenic bacteria were able to induce HR on *N. benthamiana*. Suspensions of bacteria (~10^6^ CFU/mL) were treated with serial concentrations of ZM-804 peptide (0.2 to 2 µM). At 48 h post infiltration, the leaves of *N. benthamiana* were examined and observed for HR symptoms within infiltrated sites. The lowest or minimum concentrations of ZM-804 peptide that prevented HR symptoms on *N. benthamiana* leaves (right side) were 2, 2, 1, and 0.5 µM for *R. solanacearum, P. syringae* pv. syringae, *P. syringae* pv. tomato DC3000, and *E. amylovora*, respectively. In contrast, HR symptoms were observed and photographed in the control side of the leaves (left side) under normal and UV light, respectively (Figure 5).

### 2.7. Tomato Plant Infection Assay

#### Infection of *P. syringae* pv. Tomato DC3000 on Tomato

**Figure 6:**
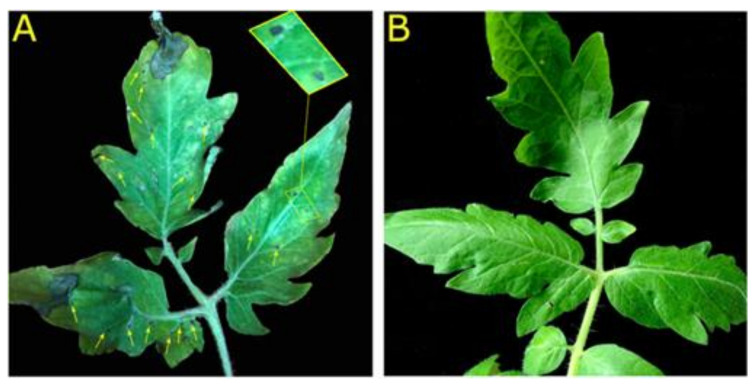
Leaf spot disease on *S. lycopersicum* prevented by ZM-804 peptide. Bacterial cells of *P. syringae* pv. tomato DC3000 **(**∼10^6^ CFU/mL) were treated with 4 µM ZM-804, while the control was treated with dd water (without peptide). In each treatment, 10 mL was sprayed directly on tomato plant leaves until both adaxial and abaxial surfaces were uniformly wet using a sprayer. Six days after inoculation, the symptoms of leaf spot disease were observed on the leaves surface of the control; yellow arrows indicate the spots in the control (**A**), while the treatment with ZM-804 prevents leaf spot of tomato caused by *P. syringae* pv. tomato DC3000 (**B**).

Based on the virulence and ability of *P. syringae* pv. tomato DC3000 to infect tomato leaves and cause leaf spot disease, the ZM-804 peptide was added to a suspension (~10^6^ CFU/mL) of bacteria with a final concentration of 4 µM. In contrast, dd water was sprayed directly on the leaves’ surface of the control treatment. After 4 days, the symptoms of leaf spot were observed on the surface of the control plant leaves, while the leaves of the tomato plants treated with ZM-804 peptide did not show any symptoms (Figure 6). These results indicated that the ZM-804 peptide might be effective in protecting plants against bacterial pathogens.

### 2.8. Hemolytic Activity of the ZM-804 Peptide

**Figure 7:**
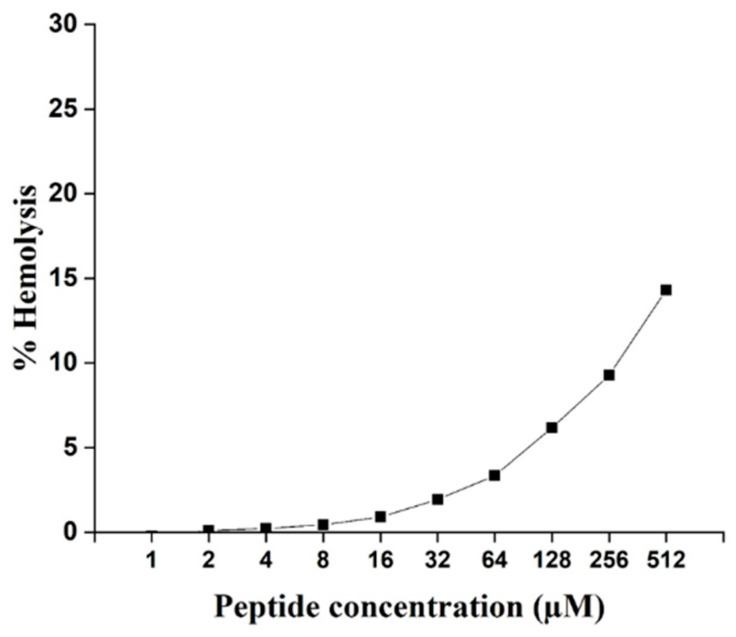
Hemolytic activity of the ZM-804 peptide. ZM-804 induced hemolysis (%) of mouse RBCs at different peptide concentrations and was defined as a ratio of complete hemolysis caused by 1% Triton X-100. Data are expressed as the means of measurement from three independent experiments.

The hemolytic activity examinations were performed against mouse red blood cells (RBCs) to investigate the effect of the ZM-804 peptide on mammalian cells. Triton X-100 (1%) for 100% hemolysis was used as a positive control and PBS buffer as a negative control. The hemolysis percentage of ZM-804 was calculated to 1% Triton X-100 and analyzed, and hemolysis started to rise after 8 µM due to 0.44% hemolysis, whereas the high concentration of 512 µM caused 14.32% hemolysis of the blood cells (Figure 7). According to the results, the ZM-804 peptide has a lower hemolytic activity at MIC values with microorganisms, which confirmed that ZM-804 peptide caused 10% hemolytic activity when the concentration is 256 µM. Our results conclude that ZM-804 peptide is not toxic to mouse RBCs in low concentration and is relatively safe concerning mammalian cells.

## 3. Discussion

Fungi, bacteria, and viruses such as pathogens can dramatically influence most of the crops’ yield and quality, which can have massive economic consequences. Mainly chemical pesticides are used to control phytopathogenic microorganisms. The management of plant pathogens is therefore critical not only economically but also for human health. Several countries have made legal reforms to limit applications of some pesticides intending to have certain compounds which are more selective, less toxic, and safer on the environment [29]. In AMPs databases (APD3), there are approximately 3000 natural antimicrobial peptides isolated from various organisms [14].

Plant genomes hold a wide range of genes for detecting pathogen attacks and inducing adequate defense responses [7,30]. *Z. mays* L. inbred line B73 is regarded as a useful resource for the isolation of antimicrobial peptides. A 33-residue low molecular weight AMP has been isolated from seeds of the maize inbred line B73 [8]. A single R gene, *Rxo*1, is regulated by a nonhost resistance response in maize to *X. oryzicola* pv. oryzicola pathogen in rice, and the R gene is able to regulate a diverse array of cellular events. After the infiltration of *X. oryzicola* pv. oryzicola which contains the corresponding *avrRxo*1 gene, the maize line B73 exhibited strong HR. The bacterial effector protein type III can trigger a strong HR in maize B73 (*Rxo*1) encoded by the *avrRxo*1 gene [31].

In this respect, this study considered the *Z. mays* inbred line B73 as a good source for the isolation and characterization of new natural antimicrobial peptides. Therefore, it was chosen for constructing the cDNA library in our study. All the clone insertions after sequencing were identified and confirmed that the source belongs to the *Z. mays* L. B73 based on the BLAST analysis.

Bioinformatic predictive methods can be suitable for larger scanning and detection of possible novel AMPs. Many computational servers are based on different features for predicting and discovering new AMPs by using several algorithms [32]. Therefore, several servers were used for high accuracy prediction of potent AMPs. According to the CAMP_R3_ server prediction results, all of the amino acid sequences that have the highest-values prediction were compared. Among all of them, the ZM-804 peptide showed the highest values. In another database, ADAM, the ZM-804 showed the highest predictive value of AMP.

As a result of physicochemical properties, the APD3 database indicated that the composition of the ZM-804 peptide is of 20 amino acids, and as a short peptide it is composed of seven different amino acids and a molecular weight (MW) of 2.569 kDa. The presence of cysteine in the composition of ZM-804 increases the possibility of the existence of a dimer peptide form. This leads to the high stability of ZM-804 against proteases and increases the selectivity on prokaryotic cells. The ZM-804 falls in the range for the length of most peptides isolated from plants (natural anti-microbial peptides) as AMPs, which is between 10 to 50 aa with a range size of MW 2 to 9 kDa [33]. In general, AMPs are composed of short sequences of 10 to 100 aa (<10 kDa) with high membrane activity and are amphipathic [34]. The simple short sequence of AMPs can reduce synthesizing costs of the peptide in application [35]. However, the AMP structure is essential, and its antimicrobial activities depend on charge, size, hydrophobicity, amphipathicity, and solubility [9].

The positive net charge plays a vital role in AMP activity. In this regard, the ZM-804 peptide has a (+4) net positive charge. The range of the net charge for most AMPs is from +4 to +6, which reflects the ideal biological activity charge [18]. Positive net charge residues connect with the lipid-peptide surface in the cell membrane, which forms close electrostatic connections with the phospholipid membranes (negative charge) of bacteria [36].

The hydrophobicity of peptides is also a main bioactivity parameter. The ratio of hydrophobic residues in a peptide is known as hydrophobicity, which is about 50 percent for AMPs [36]. The ZM-804 peptide has a 70% hydrophobicity ratio which represents a high level of hydrophobicity. Typically, AMPs contain more residues of positive charge from the hydrophilic side that can be attached to the surface of the microorganism membrane, while the hydrophobic side causes AMPs to bind into the bilayer lipid of the cell membrane to contribute to membrane depolarization and cell death by barrel-stave pore, worm-hole pore, or carpet type [37].

Peptides that have a positive net charge in the polar face can interact more efficiently with the cell membrane by binding the negatively charged side chains of phospholipids [38]. Additionally, the hydrophobic residues can integrate the nonpolar faces of AMPs into the cell membrane [39]. Hence, this feature of the helical wheel allowed the ZM-804 to acquire the amphipathic conformation. Moreover, the Boman index’s positive value (protein-binding potential) of the ZM-804 (0.95 kcal/mol) improves its potential to bind to the proteins of the bacterial cell membrane.

The alpha-helical structure was predicted for the ZM-804 peptide with an alpha-helix secondary structure, and the alpha-helical conformation was closely related to the peptide activity [40]. Several AMPs with an alpha-helix structure were already reported in previous studies [41,42]. Alpha-helical structure formation boosts the interaction between AMPs and cell membranes and improves the membrane’s permeability to the AMPs. Moreover, the physicochemical properties of the ZM-804 are mostly similar to G17 and G19 peptides in previous studies [43].

The MolProbity results showed that the ZM-804 peptide structure parameters stayed within the ranges of acceptable consistency and stability [28]. Additionally, the PROSAII-web indicated that the ZM-804 3D model was inside the structure’s favorable area, and these results were similar to earlier studies [26].

According to the BLAST results, ZM-804 showed no significant similarities with most of the other AMPs in databases. This indicates that the ZM-804 is a new natural AMP recovered from the plant of the maize inbred line B73. In silico results of the prediction analysis and the physicochemical properties of ZM-804 make it a promising AMP candidate.

In the present study, we confirmed the in silico results of ZM-804 experimentally. The ZM-804 was synthesized using the solid-phase peptide synthesis by the GenScript^®^ Corporation (Piscataway, NJ, USA) [44].

The MIC and MBC values indicated that ZM-804 has high antimicrobial activity against tested bacterial indicators (Table 2) and exhibited different effects on Gram-positive and Gram-negative bacteria. The lowest MIC values were found to be 8 and 4 µM against Gram-positive and Gram-negative bacteria, respectively. In contrast, the highest MIC value was found to be 16 µM for both Gram-positive and Gram-negative bacteria. The lowest MBC values were found to be 16 and 8 µM against Gram-positive and Gram-negative bacteria, respectively, and the highest value was found to be 32 µM for both Gram-positive and Gram-negative bacteria. Therefore, there is a distinct difference in the effectiveness of ZM-804 peptide on both bacterial groups, whereas Gram-negative bacteria are more sensitive than Gram-positive bacteria. ZM-804 agrees with the SM-985 peptide [45] in preference for Gram-negative bacteria, although they have no similar composition. Moreover, the MICs and MBCs of the ZM-804 are similar and close to previous studies on other AMPs such as NCR peptides [46,47]. Additionally, Most of the MBC values were higher than the MIC one to two potencies [46]. The MIC 50, MIC 90, and MBC were determined for G17 and G19 peptides against *E. coli* and MRSA to evaluate the antimicrobial activity [43]. This difference may be attributed to the different cell membrane compositions for each of these bacterial groups. The behavior and ability of AMPs to cross the layers of peptidoglycan and LPS in the membrane to reach the cytoplasm may contribute to the difference in values of MIC and MBC between both bacterial groups [48,49].

The MLC is defined as the value of minimum dilution of the ZM-804 that directly kills bacteria in cell suspensions of 10 mM sodium phosphate buffer for all bacterial indicators (Gram-positive and Gram-negative) which were tested [50]. The results of the MLC (4 µM) of ZM-804 were observed against bacterial indicators (Figure 2). Interestingly, the MLC values were lower than the MIC and MBC values. The main cause for the difference detected may be related to the environment’s nature where the antimicrobial activity was tested. The Muller Hinton Broth (MHB) growth medium contains a high concentration of divalent cations which can lead to inhibition of the activity of AMPs [47], whereas there are no divalent cations in the 10 mM sodium phosphate buffer (pH 7.0) used in the MLC process test. Numerous natural AMPs are already isolated, but few AMPs show potential antimicrobial activity since components such as salts and divalent cations inhibit their bioactivity [51]. Cationic AMP are linked with the negative charge bacterial cell membrane by electrostatic interactions. Na^+^, Mg^+2^, and Fe^+3^ are cations that inhibit the electrostatic interaction among the bacterial membranes and peptides, leading to a reduction in antimicrobial activity [52].

The results observed by the confocal laser-scanning microscope, shown in Figure 3, indicate that the critical target of the ZM-804 peptide is the cell membrane of living bacteria and that it showed affinity linkage with it. The localization of the FITC-labeled ZM-804 peptide is associated with the cells for all of the bacterial indicators treated by 10 µM, and these results are in agreement with other research of cationic-AMPs such as MSI-78 and MSI-594 peptides [53,54]. As a result of antimicrobial activity, the FITC tag affects the activity of the ZM-804 peptide. The lowest concentration of the peptide has no visible growth of colony on plates against *C. michiganesis* subsp. michiganesis, *C*. *fangii*, *X*. *campestris* pv. holcicola, and *X. oryzae* pv. oryzae increased to 10 µM and reduced the antimicrobial activity of FITC-ZM-804 peptide as compared with the MLC results of ZM-804. The activity of peptide NCR035 decreased after being labeled by FITC, and it was measured using PI uptake [55]. However, the FITC tag did not affect the antimicrobial activity of the SM-985 peptide [45].

Scanning and transmission electron microscopy confirmed the morphological deformation and abnormality of the cell membranes and cytoplasm of *P*. *syringae* pv. tomato DC3000 and *C*. *michiganensis* subsp. michiganensis bacterial indicators which were treated by 4 μM of the ZM-804 peptide. SEM was applied to image the effects of the ZM-804 peptide, and it is suitable to have deep visualization into the effect of antimicrobial peptides on membrane destruction and cell killing of microbes. The main target of a thanatin peptide is a component of the cell wall in Gram-positive bacteria [56]. In our Figure 3, it is shown that the FITC-labeled ZM-804 peptide is bound to bacterial cells that may involve not only the plasma membrane but also the outer membrane LPS of Gram-negative bacteria and the cell wall peptidoglycan of Gram-positive bacteria. Morphological changes also observed in SEM and TEM point to the potential binding of the peptide with the LPS outer membrane. Blisters and irregular-wrinkled cell membrane surfaces were observed as a result of the ZM-804 peptide penetration [47]. Such external membrane destabilization facilitates the penetration of cationic-AMPs and contributes to the local interruption or destruction of the inner membrane as the cytoplasmic content reaches the periplasmic space and fills it locally. Consequently, blisters form without damaging the outer membrane [57].

TEM has shown several frequently spaced protrusions on the cell membrane surface which became rough. The cytoplasmic content was not clear enough to distinguish the cell content. SEM and TEM studies have demonstrated that dysfunction of the cell membrane can contribute to ion and metabolite leakage, depolarization, and finally cell death. These results show that the ZM-804 peptide can kill Gram-positive and Gram-negative bacteria via disrupting the cell membrane, and this is in agreement with most of the previous studies [21,53,58].

One characteristic of pathogenic strains of *R. solanacearum, P. syringae* pv. syringae, *P. syringae* pv. tomato DC3000, and *E. amylovora* is that they induce HR by infiltration on leaves of *N. benthamiana*. The ZM-804 peptide was sufficient to prevent the pathogenicity of strains and impair their virulence at 2, 2, 1, and 0.5 µM for each indicator bacteria, respectively. During the HR, phenolic compounds are released, which can be visualized under UV light as yellow/green programmed cell death spots. The results of antibacterial activity in planta on *N. benthamiana* confirmed the MLC results of the ZM-804 peptide. Moreover, the ZM-804 peptide prevents *P*. *syringae* pv. tomato DC3000 to infect *S. lycopersicum* plant and cause leaf spot disease without incubation period with the ZM-804 peptide. The generation time of *P*. *syringae* pv. tomato DC3000 was reported to be ~2.5 h at 4–8 h after inoculation in tomato apoplast extracts [59], and the invasion of bacterial spraying on leaves was determined after 24 h [60]. Therefore, the ZM-804 peptide has enough time to interact with the cell membrane of Pst DC3000 and disrupt it, which leads to cell death in the end. AMPs have gained a lot of interest in plant protection as a control source with a potentially reduced negative environmental effect and a wide range of activities [61]. In general, the ZM-804 showed strong antimicrobial activity in planta, which is similar to the SM-985 peptide [45] and the VG_1_6KRKP peptide, which have significant antimicrobial activity against *Xanthomonas oryzae* and *Xanthomonas campestris*-caused disease in rice [62].

The ZM-804 peptide has lower hemolytic activity against mouse erythrocytes at MIC values. It is a common feature of cationic AMPs to cause high hemolytic activity due to their positive net charge. However, the distribution of positive charges with high hydrophobicity may lead to low hemolysis at a low peptide concentration [63]. Previous study showed that peptides with a positive charge from +4 to +8 can cause low hemolytic activity, while +9 and +10 increase hemolytic activity [64].

## 4. Materials and Methods

### 4.1. Bacterial Strains and Growth Conditions

Seeds of the maize (*Z. mays* L.) inbred line B73 were grown in pots of peat moss compost soil maintained in a growth chamber under the following conditions: 28 °C/25 °C, constant relative humidity 60%, and a photoperiod of 16 h light to 8 h dark [65]. Tobacco (*N. benthamiana*) plants were grown in nutrient-rich soil after pre-germination under greenhouse conditions for 5 weeks with a photoperiod of 14/10 h light and dark intervals at 24 °C [66], while tomato (*Solanum lycopersicum*) plants incubated for 6 weeks at 28 °C. Seeds of maize, tobacco, and tomato were provided by Key laboratory, Huazhong Agricultural University, Wuhan, China. All of the plants were grown in a growth room with an environmental control condition without fertilization.

*Rhizoctonia solani* AG-1-IA was cultured on plates of potato dextrose agar (PDA) medium and incubation for 2 days at 28 °C [65]. The plant pathogenic microbes used in this study as bacterial indicator strains are the Gram-positive strains of *C. michiganensis* subsp. michiganensis and *C. fangii* grown on a nutrient agar (NA) medium. Moreover, the following Gram-negative bacteria were also used in this study: *R. solanacearum*, *P. syringae* pv. tomato, *P. syringae* pv. syringae, *X. oryzae* pv. oryzae, *X. campestris* pv. holcicola, *D. zeae*, *P. carotovorum* subsp. carotovorum, *E. amylovora*, and *A. vitis*. The genus of *pseudomonas*, *Dickeya*, *Pectobacterium*, *Erwinia*, and *Agrobacterium* were grown on plates of King’s B (KB) medium, while the genus of *Ralstonia* and *Xanthomonas* on Luria-Bertani (LB) medium, and all of the pathogenic bacteria incubated at 28 °C. Three model strains of nonpathogenic bacteria were used: *B. subtilis* 168 as Gram-positive, and *E. coli* DE_3_ and DH_5_α as Gram-negative which were grown on LB medium and incubated at 37 °C (Table S1).

### 4.2. Construction of cDNA Library, Sequencing, and Analysis

Leaves of the *Zea mays* inbred line B73 plants (after 3 weeks of growth) were inoculated by the disk PDA of *R. solani*. Equal pieces of leaf samples were collected at different time points: 0, 6, and 12 to 96 h, collected for each 12 h, then immediately frozen in liquid nitrogen and kept at -80˚C until use. Total RNA was extracted from leaves using TRIZOL^®^ following manufacturer instructions. mRNA was purified using PolyATtract^®^ kit m-RNA isolation systems (Promega Madison, WI, USA). A cDNA library was constructed via synthesis double-strand cDNA using the PrimeScript™ double-strand cDNA synthesis kit (TaKaRa Biomedical Technology, Dalian, China) containing cleavage site *Xba* Ⅰ through Oligo (dT) primer. Three pairs of primers were added to cDNA as an adaptor (adaptors used listed in Table S2) containing cleavage site *Nde* Ⅰ [67,68]. After, ligation of cDNA fragments into the pBE-S vector was performed, then it was transformed to *E. coli* HST08, and then to *B. subtilis* SCK_6_ super-competent cells [69]. Gel electrophoresis was performed to visualize the total RNA, mRNA, and cDNA (Figure S1). The quality of the cDNA library of maize B73 was checked by PCR amplification. Colonies were chosen randomly and pBE-S primers (Table S3) were used to amplify inserts and reveal them on the gel electrophoresis (Figure S2).

The sequencing was performed for all of the colonies with an insert of more than 500 bp in length, as revealed by PCR using pBE-S primers. The program of the thermal cycle started from initial denaturation of 5 min at 95 °C, followed by 30 cycles of the following: 30 sec at 95 °C for denaturation, 30 sec at 56 °C as annealing, 1 min at 72 °C for an extension, and a final extension at 72 °C for 10 min. To evaluate the cDNA sequences, the product of PCR was sent for sequencing using the Sanger sequencing. The Basic Local Alignment Search Tool (BLAST) was used on NCBI (https://blast.ncbi.nlm.nih.gov/Blast.cgi; accessed on 1 October 2019) and Maize GDB (https://www.maizegdb.org/; accessed on 1 October 2019) to confirm the sequences of cDNA library data and their position on a chromosome of the *Z. mays* B73 genome database [70]. The cDNA sequences have been translated into amino acid sequences by a translation tool (https://web.expasy.org/translate/; accessed date: 1 October 2019) based on the codon of each amino acid according to the NCBI codes [71].

### 4.3. Bioinformatics Analysis

#### 4.3.1. In Silico Prediction for AMPs 

Computational prediction methods were used to align and screen the cDNA library of maize B73. For AMP prediction, several prediction servers were used to screen amino acid sequences selected and uploaded in FASTA format. Two servers were used for general prediction, the Collection of Anti-Microbial Peptides (CAMP_R3_) (http://www.camp.bicnirrh.res.in/index.php; accessed on 1 October 2019) [13] and the Database of Anti-Microbial Peptides (ADAM) (http://bioinformatics.cs.ntou.edu.tw/ADAM/tool.html; accessed on 1 October 2019) [72]. CAMP_R3_ (>0.5) and ADAM (>1) were used to predict AMP probability [73]. Moreover, specific prediction servers for AMPs were used to classify AMPs based on their biological activity against microbial organisms: the dbAMP (http://140.138.77.240/~dbamp/predict.php; accessed on 1 October 2019) predicts antibacterial peptide [12]; the ClassAMP (http://www.bicnirrh.res.in/classamp/predict.php; accessed on 1 October 2019) [74], the Antibp (https://webs.iiitd.edu.in/raghava/antibp/submit.html; accessed on 1 October 2019) [75], and the iAMPpred (http://cabgrid.res.in:8080/amppred/server.php; accessed on 1 October 2019) [76] for the classification of AMPs (anti-bacterial, anti-fungal, and anti-viral) based on features sequences.

#### 4.3.2. Physiochemical Characterization of the ZM-804 Peptide

The antimicrobial peptide database (APD3) (http://aps.unmc.edu/AP/prediction/prediction_main.php; accessed on 1 October 2019) and HeliQuest servers were used to predict the physicochemical properties of the ZM-804 peptide. The APD3 [14] server predicted the amino acid composition, total hydrophobic ratio, net charge, molecular weight, and Boman index. The HeliQuest (https://heliquest.ipmc.cnrs.fr/; accessed on 1 October 2019) [77] server was used for the analysis of helix types of amino acid composition and was used to calculate the hydrophobicity (<H>), hydrophobic moment (μH), and net charge (z). In the analysis, a 20-residue window was used, and for the sequence under inspection the highest discrimination factor (D) was calculated by the following equation: D = 0.944 (<μH>) + 0.33 (z). When the D is over 0.68 and <H> over 0.75, the region can be predicted to be a lipid-binding region using an algorithm Eisenberg plot methodology for identifying the hydrophobic polypeptide sequence region which discriminates between surface-study and transmembrane regions [78]. Prot pi (https://www.protpi.ch/Calculator/ProteinTool; accessed on 1 October 2019) was used for calculating the isoelectric point for proteins and peptides. The I-TASSER (https://zhanglab.ccmb.med.umich.edu/I-TASSER/; accessed on 1 August 2020) [79] and the PEP-FOLD3 (https://bioserv.rpbs.univ-paris-diderot.fr/services/PEP-FOLD3/; accessed on 1 August 2020) [80] servers were used to predict the secondary structure of the ZM-804 peptide. Three different antimicrobial peptides databases were used for blast and alignment to determine the similarities of the ZM-804 peptide: CAMP_R3_ [13], APD3 [14], and ADAM [72].

#### 4.3.3. Prediction of 3D Structure

The HeliQuest [77] was used to design the helical wheel diagram. On the other hand, the I-TASSER [79] and the PEP-FOLD 3 [80] were used to predict the 3D structure of the ZM-804 peptide and visualized by the UCSF Chimera 1.14rc program. The PROSAII [81] and MolProbity [28] web-tools were used to validate the 3D structure of the ZM-804 peptide.

### 4.4. Synthesise of the ZM-804 Peptide

The ZM-804 (LARLRRLCFLWAAAWPWPWR) peptide was synthesized in a 9-fluorenyl methoxycarbonyl (Fmoc) solid-phase method by GenScript^®^ Corporation (Piscataway, NJ, USA). Synthesized ZM-804 peptide was purified by high-performance liquid chromatography (HPLC) to a 97.4% purity, and its molecular weight was established by mass spectrometry. The concentration of ZM-804 peptide was estimated from the molecular weight (molarity) and resuspended in ultrapure water that was recommended in the test report of peptide solubility which was provided by the company, then stored at −80 °C.

### 4.5. Antimicrobial Assay of the ZM-804 Peptide

#### 4.5.1. Measuring the MIC and MBC of the ZM-804 Peptide

The antimicrobial activity assay of the ZM-804 peptide against 11 indicators of phytopathogenic bacteria along with one model strain of *B. subtilis* and two strains of *E. coli*, Gram-positive bacteria (C. *michiganesis* subsp. michiganesis, *C. fangii*, and *B*. *subtilis* 168) and Gram-negative bacteria (*R. solanacearum, P. syringae* pv. tomato DC3000, P. syringae pv. syringae, *X. oryzae* pv. oryzae, *X. campestris* pv. holcicola, *D. zeae*, *P. carotovorum* subsp. carotovorum, *E. amylovora*, *A. vitis,* and *E. coli* DE_3_ and DH_5_α) was performed to determine the MICs [82] and MBCs [50] using the broth and agar dilution method, as previously described [45]. The ZM-804 peptide was dissolved and diluted to reach a final concentration stock of 256 μM. Eight serial dilutions of the ZM-804 peptide (256, 128, 64, 32, 16, 8, 4, and 2 μM) were made in a flat-bottom Microtiter^®^ plate (96-well) in Mueller-Hinton broth (MHB) medium. The logarithmic phase of each indicator’s bacterial culture was washed by 10 mM sodium phosphate buffer (pH 7.0) and resuspended in MHB to achieve turbidity of bacterial suspension concentration of approximately 10^6^ colony-forming units per ml (CFU/mL) as measured by Spark^®^ Multimode Microplate Reader. Preparation of the bacterial indicator’s suspensions is described in detail (see Supplementary Methods). Bacterial suspensions were used to inoculate each well containing the peptide solution as well as the positive control (peptide free). The final concentration of the bacterial cells was ∼10^5^ CFU/mL. Negative control (without bacteria nor ZM-804) was performed in a separate well. Microtiter plates were incubated for 8 h at a suitable condition for all of the bacterial indicators at 28 °C or 37 °C as described above. Serial dilutions were made for the different concentrations of ZM-804 peptide, positive, and negative controls. An aliquot of 100 µl (from the suitable dilution) was plated on agar plates with a suitable medium and incubated in the same conditions. Results were observed when the colonies of the bacterial growth control were grown on the agar media. MICs were determined as the lowest concentration of ZM-804 peptide, which led to the inhibition of 80% growth of bacterial control [83]. In contrast, MBCs were determined as the minimum concentration of ZM-804 peptide that led to zero (no visible) growth of the colony on an agar plate [84]. This experiment was performed in three independent replicates, involving both positive and negative controls.

#### 4.5.2. Measuring the MLC of the ZM-804 Peptide

To determine the MLC of ZM-804 Peptide, the logarithmic phase of 14 indicator bacteria was prepared, washed, and adjusted to ∼10^6^ CFU/mL for each bacterium using 10 mM sodium phosphate buffer [85,86]; details are described in Supplementary Methods. Different final concentrations of the ZM-804 peptide at 128, 64, 32, 16, 8, 4, 2, and 1 μM were added and mixed with the bacterial suspension in micro-tubes and marked as treatments, while the dd water was added to the bacterial suspension in micro-tubes and marked as control. All of the controls and treatments of bacterial indicators were incubated for 4 h (with gentle inversion every 1 h) at 28 °C for pathogenic bacteria and 37 °C for non-pathogenic bacteria. Serial dilutions of each bacterial indicator were performed after the incubation period for treatment and control independently. Aliquots 100 µL of suitable serially diluted (30–300 CFU each plate) were plated out. For pathogenic and non-pathogenic bacterial indicators, the plates were incubated at 28 and 37 °C, respectively, until visible colonies were grown. The MLC was measured as the lowest concentration of ZM-804 peptide that could kill the bacteria cells with no visible growth of the colony on the plates. The mean value of the results was observed three times independently.

### 4.6. FITC-Labeled ZM-804 Peptide 

Fluorescein isothiocyanate (FITC)-labeled ZM-804 peptide was synthesized by GenScript^®^ Corporation (Piscataway, NJ, USA), and aminohexanoic acid (Ahx) as an alkyl spacer was used to generate it. As recommended by the company for the ZM-804 peptide, the FITC-labeled ZM-804 peptide was dissolved, considering the difference in molecular weight, and also used to determine the interaction between the ZM-804 peptide and 14 indicators of bacterial plasma (cytoplasmic) membrane [53]. Like the MLC assay, the bacterial suspensions (∼10^6^ CFU/mL) were prepared using 10 mM sodium phosphate buffer, then incubated with 10 μM FITC-labeled ZM-804 peptide for 4 h in the dark. The bacteria cells were washed twice with 10 mM sodium phosphate buffer (pH 7.0) following incubation with FITC-labeled ZM-804 peptide to remove and avoid the remaining residuals of the peptide, then it was resuspended in the same buffer. Localization of the FITC-labeled ZM-804 peptide was observed by Olympus BX61 confocal laser-scanning microscope to visualize the FITC fluorescence [21]. The 488-nm-wavelength laser for the excitation and the emission at 500–530 nm was used to detect the FITC.

To measure the effectiveness of the FITC tag on the antimicrobial activity of the ZM-804 peptide, a cell-killing examination was performed on *X*. *campestris* pv. holcicola, *X*. *oryzae* pv. oryzae, *C*. *michiganesis* subsp. michiganesis, and *C*. *fangii*. The bacterial indicator suspensions (∼10^6^ CFU/mL) were treated with different final concentrations of FITC-ZM-804 peptide at 30, 20, 10, and 5 μM in micro-tubes and marked as treatments, while the controls were treated with dd water. All of the controls and treatments were incubated for 4 h (with gentle inversion every 1 h) in the dark at 28 °C. Serial dilutions of each bacterial indicator were prepared after the incubation period for treatment and control independently. Aliquots 100 µL of suitable serially diluted (30–300 CFU each plate) were plated out for both controls and treatments and all of the plates were incubated at 28 °C. The results were measured when the visible colonies were grown on the control plates, and it was performed three times independently.

### 4.7. Observation of the Effects of the ZM-804 Peptide on Bacterial Cell Membranes

#### 4.7.1. Scanning Electron Microscopy (SEM)

The effect of the ZM-804 peptide on bacterial plasma membrane morphology was measured by scanning electron microscopy (SEM). The mid-logarithmic phase of *P. syringae* pv. tomato DC3000 and *C. michiganensis* subsp. michiganensis was used as a representative of Gram-negative and Gram-positive bacterial indicators, respectively. Briefly, both of the bacterial cell suspensions (~10^7^ CFU/mL) were washed twice and resuspended in 10 mM sodium phosphate buffer (pH 7.0). Then, they were treated with 4 μM ZM-804 peptide as treatments, and the control was treated with dd water; both treatment and control were incubated for 4 h (with gentle inversion every 1 h) at 28 °C. After incubation, both treatments and controls for each bacterial cell were pelleted and washed three times with 10 mM sodium phosphate buffer, then prepared for SEM according to [47,87] with some modifications. The bacteria cells were collected and fixed by 2.5% (*v*/*v*) glutaraldehyde solution in 10 mM phosphate buffer at 4 °C for 2 h. Then, they were washed again with 10 mM sodium phosphate buffer (three times). The bacteria cells’ pellet was dehydrated sequentially with 30, 50, 70, 90, and 100% ethanol, and air-dried for 30 min followed by lyophilization for 24 h in a freeze dryer [88]. Finally, the bacterial cell samples were coated with gold after lyophilization to avoid charging effects in the microscope and were observed under ultra-high-resolution (HITACHI SU8010) scanning electron microscope.

#### 4.7.2. Transmission Electron Microscopy (TEM)

Both *P. syringae* pv. tomato DC3000 and *C. michiganensis* subsp. michiganensis bacteria were treated with 4 μM ZM-804 peptide, followed by the same procedure as described above for SEM. After incubation, pellets of both bacterial cells were obtained and fixed with 2.5% (*v*/*v*) glutaraldehyde. The prepared bacterial cell samples were sent to Huazhong agricultural university (HZAU) center of TEM (Wuhan, China) for preparation, and a HITACHI H-7650 transmission electron microscope was used to observe the bacterial cells.

### 4.8. In planta Antimicrobial Activity Assay of the ZM-804 Peptide

#### Hypersensitive Reaction (HR) test on *N. benthamiana*

The virulence of four bacterial strains, *R. solanacearum*, *P. syringae* pv. tomato DC300, *P*. *syringae* pv. syringae, and *E. amylovora* was tested by hypersensitive reaction (HR) by infiltration in the leaves of *N. benthamiana*. Bacterial suspensions of each bacteria were collected and pelleted from the overnight culture, then cells were washed and resuspended in 10 mM sodium phosphate buffer pH 7.0, and the concentrations were adjusted to ~10^6^–10^7^ CFU/mL. A volume of (500 µL) of bacterial suspensions was treated with ZM-804 peptide at the final concentrations of 0.2, 0.5, 1, and 2 µM, and the control was treated with dd water. Both the treatment and control were incubated at 28 °C (with gentle inversion every 1 h) for 4 h. Leaves of *N. benthamiana* were cut and collected from tobacco plants grown in a growth room at 24 °C with 14/10 h light/dark for 5 weeks. A volume of 50 µL of each bacterial suspension (control/treatment) was infiltrated into distinct areas of the same leaf blade ~1–2 cm^2^ of the abaxial surface side using a plastic syringe without a needle. Symptoms of hypersensitivity responses were revealed after 24 to 48 h, then data and images were obtained through observing the tobacco leaves under normal or bright light and UV light [67]. This experiment was performed three times independently.

### 4.9. Tomato Plant Infection Assay

#### Infection of *P. syringae* pv. Tomato DC3000 on Tomato

Five-week-old seedlings of tomato (*Solanum lycopersicum*) plants were grown under a 14/10 h light/dark photoperiod at 28 °C in the growth room. A bacterial suspension of *P. syringae* pv. tomato DC3000 was prepared as described previously and we adjusted the concentration to ~10^6^ CFU/mL. The ZM-804 peptide was added to the bacterial suspension at a final concentration of 4 µM, while dd water was added to the control. Then, 10 mL of bacterial suspensions (control and treatment) was sprayed directly on the tomato plant’s surface areas until both of the surfaces were uniformly wet. After inoculation, plants (control and treatment) were covered with a nylon bag to obtain a relative humidity around the plants of 100%, and they were incubated in a growth room with a photoperiod of 14/10 h light/dark at 28 °C [45]. Six days later, the symptoms of leaf spot disease were observed, and images were obtained. This experiment was performed in triplicates independently.

### 4.10. Hemolytic Assays

Hemolytic activity assay was carried out using mouse blood cells, and 1% Triton X-100 lysis of 100% as a control agent. Two milliliters of mouse blood were collected from a healthy mouse in a tube containing ethylenediaminetetraacetic acid disodium salt and centrifuged at 700× *g* for 5 min. The pellet was resuspended and washed three times by 10 mM phosphate buffer pH 7.0, and 1% of hematocrit was prepared in the same buffered saline. Hematocrit cells were incubated with an equal volume of the serial dilutions of the ZM-804 peptide (512, 256, 128, 64, 32, 16, 8, 4, 2, and 1 μM) for 1 h at 37 °C. Hematocrit cells were incubated with 1% Triton X-100 and were considered positive control (100% lysis) [89], while incubated with phosphate buffer as a negative control. Following incubation, the hematocrit cells were centrifuged at 700× *g* for 10 min, and the absorbance of the supernatant was measured at an OD 540 nm using a microplate spectrophotometer (TECAN Spark^®^ Multimode Microplate Reader). The experiment was done three times independently, and the following equation was used to calculate the hemolytic activity percentage:

Hemolysis % = [Abs (Sample) – Abs (Neg-Avg)]/[Abs (Pos-Avg) – Abs (Neg-Avg)] × 100%

Abs (sample) = absorbance of the samples, Abs (Neg-Avg) = averaged absorbance of the negative controls, and Abs (Pos-Avg) = averaged absorbance of the positive controls.

## 5. Conclusions

AMPs represent a promising alternative in pathogen control. In this study, we have successfully isolated a ZM-804 peptide as a novel plant AMP. ZM-804 was predicted as an AMP after the screening of a cDNA library using in silico prediction tools. Physiochemical properties showed that ZM-804 is a promising AMP with an alpha-helical structure. ZM-804 was experimentally demonstrated to have broad-spectrum antibacterial activities with growth inhibition and killing of a wide range of Gram-negative and Gram-positive bacteria. Additionally, ZM-804 peptide is effective on non-plant pathogenic bacterial strains. Furthermore, ZM-804 has been shown to interact with the membranes (outer, inner) of bacteria, which led to an increase in cell membrane permeability, disruption of the bacterial cell structure (the cell wall peptidoglycan of Gram-positive and LPS of Gram-negative bacteria) and cell death in the end. In planta, ZM-804 prevented virulent bacteria pathogens from inducing HR symptoms on the leaves of *N. benthamiana*. Likewise, leaf spot disease infection on *S. lycopersicum* plants was prevented by the ZM-804 peptide. The peptide ZM-804 has low toxicity to mouse blood cells and can be regarded as a safe AMP for mammalian cells. Our present study provides evidence for the ZM-804 peptide as a good alternative for pathogen control. Importantly, ZM-804 can be used in gene transformation to generate a pathogen-resistant plant.

## Figures and Tables

**Table 1:** Physicochemical properties of the ZM-804 peptide.

Primary Structure	LARLRRLCFLWAAAWPWPWR
Hydrophobic amino acid	I: 0, V: 0, L: 4, F: 1, C: 1, M: 0, A: 4, W: 4
MW (Da)	2569.131
Net charge	+4
Total hydrophobic ratio	70%
Protein-binding potential (Boman index) kcal/mol	0.95
GRAVY	0.145

The physiochemical properties were predicted by two servers: the APD3 and the HeliQuest.

**Table 2:** Antimicrobial activity assay of the ZM-804 peptide. Minimal inhibitory concentrations (MIC) and minimum bactericidal concentrations (MBC) values (μM) against bacterial indicators.

Bacterial Indicators	MIC	MBC
*C. michiganensis* subsp. michiganensis	8	16
*C. fangii*	8	16
*Bacillus subtilis* 168	16	32
*R. solanacearum*	8	16
*P. syringae* pv. tomato DC3000	4	8
*P. syringae* pv. syringae	4	8
*X. oryzae* pv. oryzae	4	8
*X. campestris* pv. holcicola	4	8
*Dickeya zeae*	16	32
*P. carotovorum* subsp. carotovorum	16	32
*Erwinia amylovora*	8	16
*Agrobacterium vitis*	8	16
*E. coli* DH5α	16	32
*E. coli* DE3	16	32

MIC and MBC tests were performed in accordance with the Broth and Agar method.

## Supplementary Materials

Supplementary Materials can be found at https://www.mdpi.com/1422-0067/22/5/2643/s1. Table S1: List of bacterial strains and vectors used in this study, Table S2: The sequences of Adaptors, Figure S1: The total RNA, mRNA, and cDNA from left to right, Table S3: pBE-S DNA primers, Figure S2: Random insert sizes in the *Zea mays* cDNA library, Table S4: Antimicrobial peptide results prediction of ZM-804, Table S5: The classification of ZM-804 prediction, Table S6. The relative amount of bacterial suspension containing approximately 10^6^ CFU (OD_600_ 0.1).

## References

[1] Riedl S., Zweytick D., Lohner K. (2011). Membrane-Active Host Defense Peptides—Challenges and Perspectives for the Development of Novel Anticancer Drugs. Chem. Phys. Lipids.

[2] Huang Y., Huang J., Chen Y. (2010). Alpha-Helical Cationic Antimicrobial Peptides: Relationships of Structure and Function. Protein Cell Rev..

[3] Steinstraesser L., Kraneburg U.M., Hirsch T., Kesting M., Steinau H.-U., Jacobsen F., Al-Benna S. (2009). Host Defense Peptides as Effector Molecules of the Innate Immune Response: A Sledgehammer for Drug Resistance?. Int. J. Mol. Sci..

[4] Reddick L.E., Alto N.M. (2014). Bacteria Fighting Back: How Pathogens Target and Subvert the Host Innate Immune System. Mol. Cell.

[5] Hancock R.E., Haney E.F., Gill E.E. (2016). The immunology of host defense peptides: Beyond antimicrobial activity. Nat. Rev. Immunol..

[6] Oh R., Lee M.J., Kim Y.-O., Nam B.-H., Kong H.J., Kim J.-W., Park J.-Y., Seo J.-K., Kim D.-G. (2020). Myticusin-beta, antimicrobial peptide from the marine bivalve, Mytilus coruscus. Fish Shellfish Immunol..

[7] Pan Q., Wendel J., Fluhr R. (2000). Divergent Evolution of Plant NBS-LRR Resistance Gene Homologues in Dicot and Cereal Genomes. J. Mol. Evol..

[8] Duvick J., Rood T., Rao A., Marshak D. (1992). Purification and characterization of a novel antimicrobial peptide from maize (Zea mays L.) kernels. J. Biol. Chem..

[9] Bahar A.A., Ren D. (2013). Antimicrobial Peptides. Pharmaceuticals.

[10] Weidmann J., Craik D.J. (2016). Discovery, structure, function, and applications of cyclotides: Circular proteins from plants. J. Exp. Bot..

[11] Hammami R., Ben Hamida J., Vergoten G., Fliss I. (2008). PhytAMP: A database dedicated to antimicrobial plant peptides. Nucleic Acids Res..

[12] Jhong J.-H., Chi Y.-H., Li W.-C., Lin T.-H., Huang K.-Y., Lee T.-Y. (2019). dbAMP: An integrated resource for exploring antimicrobial peptides with functional activities and physicochemical properties on transcriptome and proteome data. Nucleic Acids Res..

[13] Waghu F.H., Idicula-Thomas S. (2020). Collection of antimicrobial peptides database and its derivatives: Applications and beyond. Protein Sci..

[14] Wang G., Li X., Wang Z. (2016). APD3: The antimicrobial peptide database as a tool for research and education. Nucleic Acids Res..

[15] Usmani S.S., Kumar R., Bhalla S., Kumar V., Raghava G.P.S. (2018). In Silico Tools and Databases for Designing Peptide-Based Vaccine and Drugs. Advances in Protein Chemistry and Structural Biology.

[16] Takahashi D., Shukla S.K., Prakash O., Zhang G. (2010). Structural determinants of host defense peptides for antimicrobial activity and target cell selectivity. Biochimie.

[17] Dathe M., Schümann M., Wieprecht T., Winkler A., Beyermann M., Krause E., Matsuzaki K., Murase O., Bienert M. (1996). Peptide Helicity and Membrane Surface Charge Modulate the Balance of Electrostatic and Hydrophobic Interactions with Lipid Bilayers and Biological Membranes. Biochemistry.

[18] Giangaspero A., Sandri L., Tossi A. (2001). Amphipathic Alpha Antimicrobial Peptides. Eur. J. Biochem..

[19] Jindal M.H., Le C.F., Mohd Yusof M.Y., Sekaran S.D. (2014). Net Charge, Hydrophobicity and Specific Amino Acids Contribute to the Activity of Antimicrobial Peptides. J. Health Transl. Med..

[20] Clinical and Laboratory Standards Institute (2018). M02 Performance Standards for Antimicrobial Disk Susceptibility Tests.

[21] Farkas A., Maróti G., Kereszt A., Kondorosi É (2017). Comparative Analysis of the Bacterial Membrane Disruption Effect of Two Natural Plant Antimicrobial Peptides. Front. Microbiol..

[22] Sinha S., Zheng L., Mu Y., Ng W.J., Bhattacharjya S. (2017). Structure and Interactions of A Host Defense Antimicrobial Peptide Thanatin in Lipopolysaccharide Micelles Reveal Mechanism of Bacterial Cell Agglutination. Sci. Rep..

[23] Tolba I., Zaki M. (2011). Characterization of Agrobacterium vitis isolates obtained from galled grapevine plants in Egypt. Ann. Agric. Sci..

[24] Kannan V.R., Kannan V.R., Bastas K.K. (2015). Quorum Sensing in Plant Pathogenic and Plant-Associated Bacteria. Sustainable Approaches to Controlling Plant Pathogenic Bacteria.

[25] Daoubi M., Hernández-Galán R., Benharref A., Collado I.G. (2005). Screening Study of Lead Compounds for Natural Product-Based Fungicides: Antifungal Activity and Biotransformation of 6α,7α-Dihydroxy-β-himachalene byBotrytis cinerea. J. Agric. Food Chem..

[26] Würz J.M., Güntert P. (2017). Peak picking multidimensional NMR spectra with the contour geometry based algorithm CYPICK. J. Biomol. NMR.

[27] Sippl M.J. (1993). Recognition of errors in three-dimensional structures of proteins. Proteins Struct. Funct. Bioinform..

[28] Chen V.B., Arendall W.B., Headd J.J., Keedy D.A., Immormino R.M., Kapral G.J., Murray L.W., Richardson J.S., Richardson D.C. (2009). MolProbity: All-atom structure validation for macromolecular crystallography. Acta Crystallogr. Sect. D Biol. Crystallogr..

[29] Agrios G. (2004). Plant Pathology.

[30] Meyers B.C., Dickerman A.W., Michelmore R.W., Sivaramakrishnan S., Sobral B.W., Young N.D. (1999). Plant disease resistance genes encode members of an ancient and diverse protein family within the nucleotide-binding superfamily. Plant J..

[31] Zhao B., Lin X., Poland J., Trick H., Leach J., Hulbert S. (2005). A maize resistance gene functions against bacterial streak disease in rice. Proc. Natl. Acad. Sci. USA.

[32] Liu S., Fan L., Sun J., Lao X., Zheng H. (2017). Computational resources and tools for antimicrobial peptides. J. Pept. Sci..

[33] Nawrot R., Barylski J., Nowicki G., Broniarczyk J., Buchwald W., Goździcka-Józefiak A. (2014). Plant antimicrobial peptides. Folia Microbiol..

[34] Reddy K., Yedery R., Aranha C. (2004). Antimicrobial peptides: Premises and promises. Int. J. Antimicrob. Agents.

[35] Ong Z.Y., Cheng J., Huang Y., Xu K., Ji Z., Fan W., Yang Y.Y. (2014). Effect of stereochemistry, chain length and sequence pattern on antimicrobial properties of short synthetic β-sheet forming peptide amphiphiles. Biomaterials.

[36] Yount N.Y., Bayer A.S., Xiong Y.Q., Yeaman M.R. (2006). Advances in antimicrobial peptide immunobiology. Pept. Sci. Orig. Res. Biomol..

[37] Sitaram N., Nagaraj R. (2002). Host-defense Antimicrobial Peptides: Importance of Structure for Activity. Curr. Pharm. Des..

[38] Fernandez D.I., Lee T.-H., Sani M.-A., Aguilar M.-I., Separovic F. (2013). Proline Facilitates Membrane Insertion of the Antimicrobial Peptide Maculatin 1.1 via Surface Indentation and Subsequent Lipid Disordering. Biophys. J..

[39] He J., Luo X., Jin D., Wang Y., Zhang T. (2018). Identification, Recombinant Expression, and Characterization of LGH2, a Novel Antimicrobial Peptide of Lactobacillus casei HZ1. Molecules.

[40] Park C.B., Yi K.-S., Matsuzaki K., Kim M.S., Kim S.C. (2000). Structure—Activity analysis of buforin II, a histone H2A-derived antimicrobial peptide: The proline hinge is responsible for the cell-penetrating ability of buforin II. Proc. Natl. Acad. Sci. USA.

[41] Tam J.P., Wang S., Wong K.H., Tan W.L. (2015). Antimicrobial Peptides from Plants. Pharmaceuticals.

[42] Bonduelle C. (2018). Secondary Structures of Synthetic Polypeptide Polymers. Polym. Chem..

[43] Gómez-Sequeda N., Ruiz J., Ortiz C., Urquiza M., Torres R. (2020). Potent and Specific Antibacterial Activity against Escherichia Coli O157:H7 and Methicillin Resistant Staphylococcus Aureus (Mrsa) of G17 and G19 Peptides Encapsulated into Poly-Lactic-Co-Glycolic Acid (Plga) Nanoparticles. Antibiotics.

[44] Mäde V., Els-Heindl S., Beck-Sickinger A.G. (2014). Automated solid-phase peptide synthesis to obtain therapeutic peptides. Beilstein J. Org. Chem..

[45] Qutb A.M., Wei F., Dong W. (2020). Prediction and Characterization of Cationic Arginine-Rich Plant Antimicrobial Peptide SM-985 From Teosinte (Zea mays ssp. mexicana). Front. Microbiol..

[46] Domhan C., Uhl P., Meinhardt A., Zimmermann S., Kleist C., Lindner T., Leotta K., Mier W., Wink M. (2018). A novel tool against multiresistant bacterial pathogens: Lipopeptide modification of the natural antimicrobial peptide ranalexin for enhanced antimicrobial activity and improved pharmacokinetics. Int. J. Antimicrob. Agents.

[47] Farkas A., Pap B., Kondorosi É., Maróti G. (2018). Antimicrobial Activity of NCR Plant Peptides Strongly Depends on the Test Assays. Front. Microbiol..

[48] Yang S.-T., Lee J.Y., Kim H.-J., Eu Y.-J., Shin S.Y., Hahm K.-S., Kim J.I. (2006). Contribution of a central proline in model amphipathic α-helical peptides to self-association, interaction with phospholipids, and antimicrobial mode of action. FEBS J..

[49] Silhavy T.J., Kahne D., Walker S. (2010). The bacterial cell envelope. Cold Spring Harb. Perspect. Biol..

[50] Clinical and Laboratory Standards Institute (1999). Methods for Determining Bactericidal Activity of Antimicrobial Agents: Approved Guideline.

[51] Chang T.-W., Lin Y.-M., Wang C.-F., Liao Y.-D. (2012). Outer Membrane Lipoprotein Lpp Is Gram-negative Bacterial Cell Surface Receptor for Cationic Antimicrobial Peptides. J. Biol. Chem..

[52] Zhu X., Dong N., Wang Z., Ma Z., Zhang L., Ma Q., Shan A. (2014). Design of imperfectly amphipathic α-helical antimicrobial peptides with enhanced cell selectivity. Acta Biomater..

[53] Hong J., Guan W., Jin G., Zhao H., Jiang X., Dai J. (2015). Mechanism of tachyplesin I injury to bacterial membranes and intracellular enzymes, determined by laser confocal scanning microscopy and flow cytometry. Microbiol. Res..

[54] Bhunia A., Ramamoorthy A., Bhattacharjya S. (2009). Helical Hairpin Structure of a Potent Antimicrobial Peptide MSI-594 in Lipopolysaccharide Micelles by NMR Spectroscopy. Chem. A Eur. J..

[55] Van De Velde W., Zehirov G., Szatmari A., Debreczeny M., Ishihara H., Kevei Z., Farkas A., Mikulass K., Nagy A., Tiricz H. (2010). Plant Peptides Govern Terminal Differentiation of Bacteria in Symbiosis. Science.

[56] Dash R., Bhattacharjya S. (2021). Thanatin: An Emerging Host Defense Antimicrobial Peptide with Multiple Modes of Action. Int. J. Mol. Sci..

[57] Hartmann M., Berditsch M., Hawecker J., Ardakani M.F., Gerthsen D., Ulrich A.S. (2010). Damage of the Bacterial Cell Envelope by Antimicrobial Peptides Gramicidin S and PGLa as Revealed by Transmission and Scanning Electron Microscopy. Antimicrob. Agents Chemother..

[58] Liu W.-P., Chen Y.-H., Ming X., Kong Y. (2015). Design and Synthesis of a Novel Cationic Peptide with Potent and Broad-Spectrum Antimicrobial Activity. BioMed Res. Int..

[59] Rico A., Preston G.M. (2008). Pseudomonas syringae pv. tomato DC3000 Uses Constitutive and Apoplast-Induced Nutrient Assimilation Pathways to Catabolize Nutrients That Are Abundant in the Tomato Apoplast. Mol. Plant Microbe Interact..

[60] Cai R., Lewis J., Yan S., Liu H., Clarke C.R., Campanile F., Almeida N.F., Studholme D.J., Lindeberg M., Schneider D. (2011). The Plant Pathogen Pseudomonas syringae pv. tomato Is Genetically Monomorphic and under Strong Selection to Evade Tomato Immunity. PLoS Pathog..

[61] Morassutti C., De Amicis F., Skerlavaj B., Zanetti M., Marchetti S. (2002). Production of a recombinant antimicrobial peptide in transgenic plants using a modified VMA intein expression system. FEBS Lett..

[62] Datta A., Ghosh A., Airoldi C., Sperandeo P., Mroue K.H., Jiménez-Barbero J., Kundu P., Ramamoorthy A., Bhunia A. (2015). Antimicrobial Peptides: Insights into Membrane Permeabilization, Lipopolysaccharide Fragmentation and Application in Plant Disease Control. Sci. Rep..

[63] Yin L.M., Edwards M.A., Li J., Yip C.M., Deber C.M. (2012). Roles of Hydrophobicity and Charge Distribution of Cationic Antimicrobial Peptides in Peptide-Membrane Interactions. J. Biol. Chem..

[64] Jiang Z., Vasil A.I., Hale J.D., Hancock R.E.W., Vasil M.L., Hodges R.S. (2008). Effects of Net Charge and the Number of Positively Charged Residues on the Biological Activity of Amphipathic α-Helical Cationic Antimicrobial Peptides. Peptide Sci..

[65] Huan J., Wan K., Liu Y., Dong W., Wang G. (2013). Removing PCR for the elimination of undesired DNA fragments cycle by cycle. Sci. Rep..

[66] Yang L.Y., Yang S.L., Li J.Y., Ma J.H., Pang T., Zou C.M., He B., Gong M. (2018). Effects of different growth temperatures on growth, development, and plastid pigments metabolism of tobacco (Nicotiana tabacum L.) plants. Bot. Stud..

[67] Kong X., Yang M., Abbas H.M.K., Wu J., Li M., Dong W. (2018). Antimicrobial genes from Allium sativum and Pinellia ternata revealed by a Bacillus subtilis expression system. Sci. Rep..

[68] Wu J., Abbas H.M.K., Li J., Yuan Y., Liu Y., Wang G., Dong W. (2020). Cell Membrane-Interrupting Antimicrobial Peptides from Isatis Indigotica Fortune Isolated by a Bacillus Subtilis Expression System. Biomolecules.

[69] Li J., Islam S., Guo P., Hu X., Dong W. (2020). Isolation of Antimicrobial Genes from *Oryza rufipogon* Griff by Using a *Bacillus subtilis* Expression System with Potential Antimicrobial Activities. Int. J. Mol. Sci..

[70] Andorf C.M., Cannon E.K., Portwood J.L., Gardiner J.M., Harper L.C., Schaeffer M.L., Braun B.L., Campbell D.A., Vinnakota A.G., Sribalusu V.V. (2015). MaizeGDB update: New tools, data and interface for the maize model organism database. Nucleic Acids Res..

[71] Osawa S., Muto A., Jukes T.H., Ohama T. (1990). Evolutionary changes in the genetic code. Proc. R. Soc. B Boil. Sci..

[72] Lee H.-T., Lee C.-C., Yang J.-R., Lai J.Z.C., Chang K.Y. (2015). A Large-Scale Structural Classification of Antimicrobial Peptides. BioMed Res. Int..

[73] Grafskaia E.N., Polina N.F., Babenko V.V., Kharlampieva D.D., Bobrovsky P.A., Manuvera V.A., Farafonova T.E., Anikanov N.A., Lazarev V.N. (2018). Discovery of novel antimicrobial peptides: A transcriptomic study of the sea anemone Cnidopus japonicus. J. Bioinform. Comput. Biol..

[74] Joseph S., Karnik S., Nilawe P., Jayaraman V.K., Idicula-Thomas S. (2012). ClassAMP: A Prediction Tool for Classification of Antimicrobial Peptides. IEEE ACM Trans. Comput. Biol. Bioinform..

[75] Lata S., Sharma B., Raghava G. (2007). Analysis and prediction of antibacterial peptides. BMC Bioinform..

[76] Meher P.K., Sahu T.K., Rao A.R. (2016). Performance evaluation of neural network, support vector machine and random forest for prediction of donor splice sites in rice. Indian J. Genet. Plant Breed..

[77] Gautier R., Douguet D., Antonny B., Drin G. (2008). HELIQUEST: A web server to screen sequences with specific α-helical properties. Bioinformatics.

[78] Keller R.C. (2011). New User-Friendly Approach to Obtain an Eisenberg Plot and Its Use as a Practical Tool in Protein Sequence Analysis. Int. J. Mol. Sci..

[79] Roy A., Kucukural A., Zhang Y. (2010). I-TASSER: A unified platform for automated protein structure and function prediction. Nat. Protoc..

[80] Lamiable A., Thévenet P., Rey J., Vavrusa M., Derreumaux P., Tufféry P. (2016). PEP-FOLD3: Fasterde novostructure prediction for linear peptides in solution and in complex. Nucleic Acids Res..

[81] Wiederstein M., Sippl M.J. (2007). ProSA-web: Interactive web service for the recognition of errors in three-dimensional structures of proteins. Nucleic Acids Res..

[82] Clinical and Laboratory Standards Institute (2012). M07-A9: Methods for Dilution Antimicrobial Susceptibility Tests for Bacteria That Grow Aerobically; Approved Standard.

[83] Wu X., Wang Z., Li X., Fan Y., He G., Wan Y., Yu C., Tang J., Li M., Zhang X. (2014). In VitroandIn VivoActivities of Antimicrobial Peptides Developed Using an Amino Acid-Based Activity Prediction Method. Antimicrob. Agents Chemother..

[84] European Committee for Antimicrobial Susceptibility Testing (EUCAST) of the European Society of Clinical Microbiology and Infectious Diseases (ESCMID) (2003). Determination of minimum inhibitory concentrations (MICs) of antibacterial agents by broth dilution. Clin. Microbiol. Infect..

[85] Ribeiro C.W., Baldacci-Cresp F., Pierre O., Larousse M., Benyamina S., Lambert A., Hopkins J., Castella C., Cazareth J., Alloing G. (2017). Regulation of Differentiation of Nitrogen-Fixing Bacteria by Microsymbiont Targeting of Plant Thioredoxin s1. Curr. Biol..

[86] Saikia K., Sravani Y.D., Ramakrishnan V., Chaudhary N. (2017). Highly potent antimicrobial peptides from N-terminal membrane-binding region of E. coli MreB. Sci. Rep..

[87] Ferreira S., Silva F., Queiroz J.A., Oleastro M., Domingues F.C. (2014). Resveratrol against Arcobacter butzleri and Arcobacter cryaerophilus: Activity and effect on cellular functions. Int. J. Food Microbiol..

[88] Shi W., Li C., Li M., Zong X., Han D., Chen Y. (2016). Antimicrobial peptide melittin against *Xanthomonas oryzae* pv. *oryzae*, the bacterial leaf blight pathogen in rice. Appl. Microb. Cell Physiol..

[89] Mohanram H., Bhattacharjya S. (2016). ‘Lollipop’-shaped helical structure of a hybrid antimicrobial peptide of temporin B-lipopolysaccharide binding motif and mapping cationic residues in antibacterial activity. Biochim. Biophys. Acta Gen. Subj..

