# Supplementary material for: Prediction and Activity of a Cationic α-Helix Antimicrobial Peptide ZM-804 from Maize
Source: Int J Mol Sci. 2021 Mar 5;22(5):2643. doi: 10.3390/ijms22052643 (PMC7961353; doi:10.3390/ijms22052643)
Supplement: Supplementary file 1 [file ijms-22-02643-s001.pdf]

Supplementary Materials

Media:

Nutrient agar (NA) medium

Tryptone 10g.

Beef extract 3g.

NaCl 5g.

Agar 15g. per liter; pH 7.0.

Luria-Bertani (LB) medium

Tryptone10g.

Yeast extract 5g.

NaCl 10g.

Agar 15g. per liter; pH 7.0.

King's B (KB) medium

Protease peptone 10g.

K_2HPO_4 anhydrous 1.5g.

Glycerol 15g,

$MgSO_4$ (1 M) 5ml.

Agar 15g per liter; pH 7.0.

Sodium Phosphate Buffer 10 mM

To make 1L of 10 mM Sodium Phosphate Buffer, two stock solutions were prepared. Stock solution **A**: $Na_2HPO_4 \cdot 12H_2O$, **M.W**. 358.22; 20 mM solution containing 7.16 g/L was dissolved in dd water. Stock solution **B**: $NaH_2PO_4 \cdot 2H_2O$, M.W. 156.03; 0.2 M solution containing 3.12 g/L was dissolved in dd water. To prepare 1L of sodium phosphate buffer 10 mM pH 7.0, we added 350 ml of solution **A** to 195 ml of solution **B**, then mixed and the volume was adjusted to 1L by dd water. The buffer was autoclaved at 121°C for 20 min.

Preparation of the Bacterial Suspension:

A single colony from each bacterial indicator was grown in suitable broth media and incubated 6 to 8 h at 37°C for non-pathogenic bacteria, and 28°C for pathogenic bacteria. These starter cultures of the indicator bacteria were grown to reach OD_{600} of 0.5 to 1.0, then the pellet of 1 ml of bacterial culture was collected by centrifugation at 6000 rpm for 2 min and rinsed two times by 10 mM sodium phosphate buffer (pH 7.0). The pellet was resuspended and diluted by buffer until it reached to 0.1 of the OD_{600} (TECAN Spark® Multimode Microplate Reader). To estimate the number of colony-forming units per ml (CFU/ml) for indicator bacteria, a hemocytometer and light microscope was used to calculate the number of bacterial cells CFU/ml of each bacterial suspension at OD_{600} 0.1. To

adjust, approximately 1 × 10^6 CFU/ml, the amount of each suspension of bacteria at an OD_{600} of 0.1 containing approximately 10^6 CFU was diluted in 1 ml of buffer [1] (Table S6).

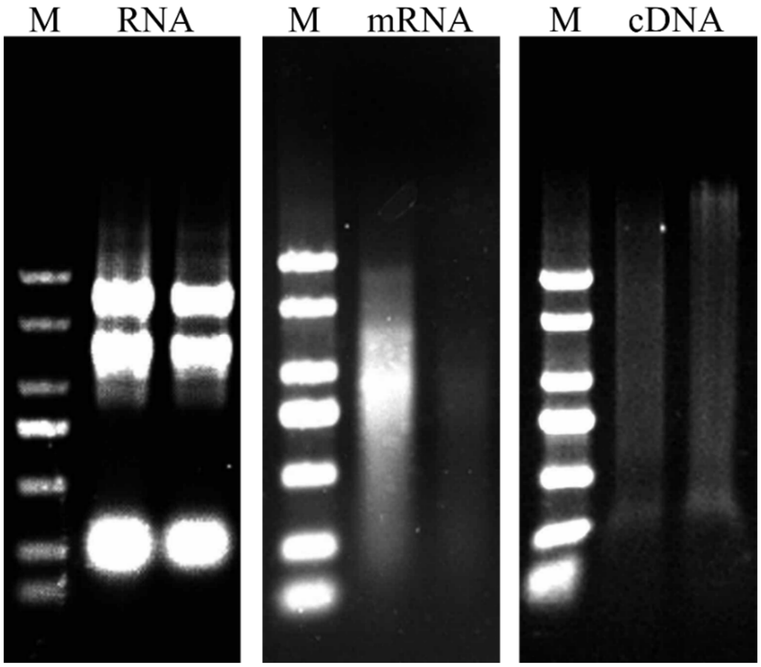

Figure S1. The total RNA, mRNA, and cDNA from left to right. The marker is DL2000.

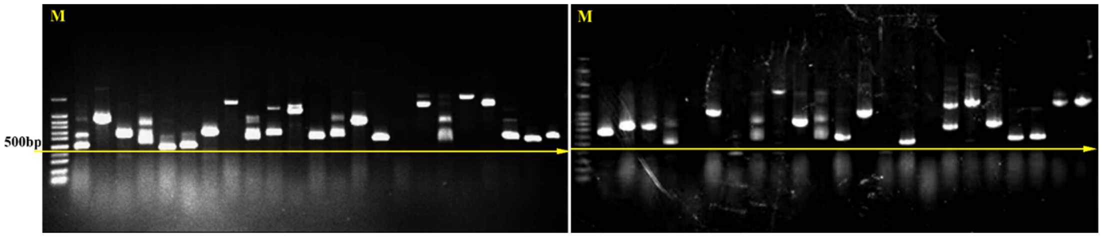

Figure S2. Random insert sizes in the *Zea mays* cDNA library. (Left) After transformation into *E. coli* HST08. (Right) After transformation into *B. subtilis* SCK_6. The yellow arrow represents the empty vector band size (500 bp). The marker in (Left) is 100 bp and (Right) DL2000.

Table 1. List of bacterial strains and vectors used in this study.

Materials	Description	Reference
**Bacterial Strains**		
Clavibacter michiganensis ssp. michiganensis YCKYB1	Anaerobic non-sporulating Gram-positive plant pathogenic bacterial	This study
Clavibacter fangii 1.1999	Wheat blight pathogen	CGMCC *
Bacillus subtilis168	*trpC2*	[2]
Ralstonia solanacearum R21-5	Soil-borne plant pathogenic bacteria	This study
Pseudomonas syringae pv. tomato DC3000	Model of Plant Pathogenic	
Pseudomonas syringae pv. syringae	Wildfire pathogenic bacteria isolated from tobacco	This study

Xanthomonas oryzae pv. oryzae XG-25	A seed-borne and transmitted pathogen can cause a serious bacterial leaf blight of rice	This study
Xanthomonas campestris pv. holcicola 1.1530	Pathogenic species on maize	CGMCC
Dickeya zeae 1.3614	Pathogenic species on maize	CGMCC
Erwinia amylovora E76	Fire blight Pathogenic bacteria isolated from pear	This study
Pectobacterium carotovorum ssp. carotovorum	Pathogenic bacteria cause rot	This study
Agrobacterium vitis	Pathogenic bacteria cause galls on grapes	[3]
Escherichia coli DE_3	Model positive Gram bacteria	
Escherichia coli DH5α	Model positive Gram bacteria	
Escherichia coli HST08	High-efficiency competent cells	This study
Plasmid pBE-S	*B. subtilis-E. coli* shuttle vector	TaKaRa

* China General Microbiological Culture Collection Center, Beijing, China (CGMCC).

Table 2. The sequences of Adaptors.

Adaptor	Sequence
1	CTCGAGAGGAATTC**CATATG**C
2	G**CATATG**GAATTCCTCTCGAGTACG
3	CTCGAGAGGAATTC**CATATG**CT
4	AG**CATATG**GAATTCCTCTCGAGTACG
5	CTCGAGAGGAATTC**CATATG**CTA
6	TAG**CATATG**GAATTCCTCTCGAGTACG

Table 3. pBE-S DNA primers.

**Forward primer**	GTTATTTCGAGTCTCTACGG
**Reverse primer**	TAACCAAGCCTATGCCTACA

The PCR protocol for the colony was started at 95°C for 5 min, followed by 28 cycles which were performed (95°C for 30 s, 56°C for 30 s, 72°C for 55 s), and then 72°C for 10 min.

Table 4. Antimicrobial peptide results prediction of ZM-804.

Name of Peptide	Peptide Sequencing	CAMP$_{R3}$ [a] SVMa1	ANNa2 DAa4	RFa3	ADAM [b]
ZM-804	LAR-LRRLCFLWAAAWPWPWR	0.722	AMP 0.950	0.949	1.34

[a] Collection of anti-microbial peptides R3 with four predicting algorithms (1- Supported vector machine, 2- Artificial neural network 3- Random forest, 4- Discriminant analysis). [b]. A Database of anti-microbial peptides.

Table 5. The classification of ZM-804 prediction.

Sever	Algorithms	AMP probability	Class (AMP/NAMP)
DbAMP	RF	1.0	Antibacteria
ClassAMP	SVM	0.972	Antifungal
	RF	0.974	Antibacterial

iAMPpred	SVM	0.51	Antibacterial
		0.33	Antiviral
		0.067	Antifungal
AntiBp	SVM	0.125	Antibacterial
	ANN	0.990	Antibacterial

Table 6. The relative amount of bacterial suspension containing approximately 10^6 CFU (OD_{600} 0.1).

Bacterial Strains	Volume (μL)
Ralstonia solanacearum	70
Pseudomonas syringae pv. tomato DC3000	75
Pseudomonas syringae pv. syringae	95
Xanthomonas oryzae pv. oryzae	90
Xanthomonas campestris pv. holcicola 1.1530	65
Dickeya zeae 1.3614	90
Erwinia amylovora	55
Pectobacterium carotovorum ssp. carotovorum	55
Agrobacterium vitis	75
Escherichia coli DE_3	100
Escherichia coli DH5α	110
Clavibacter michiganensis ssp. michiganensis	90
Clavibacter fangii 1.1999	85
*Bacillus subtilis*168	110
